# Trends in drinking habits among adolescents in the Baltic countries over the period of transition: HBSC survey results, 1993–2002

**DOI:** 10.1186/1471-2458-6-67

**Published:** 2006-03-15

**Authors:** Apolinaras Zaborskis, Linas Sumskas, Mai Maser, Iveta Pudule

**Affiliations:** 1 Institute for Biomedical Research of Kaunas University of Medicine, Eiveniu 4, LT-50009, Kaunas, Lithuania; 2 National Institute for Health Development, Hiiu 42, EE-11619, Tallinn, Estonia; 3 Health Promotion State Agency, Skolas 3, LV-1010, Riga, Latvia

## Abstract

**Background:**

The Baltic countries – Estonia, Latvia, and Lithuania – are considered to be an example of regional homogeneity over the period of transition. The World Health Organization cross-national study on Health Behavior in School-aged Children (HBSC) allows a comparison and time trends analysis of behavioral patterns among adolescents in this region. The aim of this study was to estimate the prevalence and trends of alcohol consumption and drunkenness among adolescents of Estonia, Latvia, and Lithuania in 1993/94, 1997/98, and 2001/02.

**Methods:**

Representative samples of 5286 boys and 6485 girls aged 15 from Estonia, Latvia, and Lithuania were surveyed in 1993/94, 1997/98, and 2001/02 school-year within the framework of HBSC study. The standardized survey methods were applied. The research focused on the following outcome variables: i) frequency of drinking beer, wine, and spirits; and ii) frequency of drunkenness. The same wording of questions on the consumption of alcohol was retained in each survey.

**Results:**

Beer was the most frequently used alcoholic beverage across the Baltic countries among adolescents. The rate of weekly drinking of any alcoholic beverage increased considerably during the eight years of observation, especially among Estonian and Lithuanian students. In 2001/02, 25% of boys and 12.5% of girls have reported drinking alcohol at least weekly. The rate of regular alcohol drinking was two times higher in boys, while irregular drinking was more prevalent in girls. Two or more episodes of drunkenness in the lifespan were reported by 30% of boys and 15% of girls in 1993/94 and by 52% of boys and 36% of girls in 2001/02. The use of alcoholic beverages was related to the perceived family wealth: the students from the families perceived by them as wealthy were more likely to drink weekly as compared to the students from the families perceived by them as not wealthy.

**Conclusion:**

Over the period between 1993 and 2002 the prevalence of alcohol consumption among adolescents increased considerably across the Baltic countries. The efforts of dealing with this problem should employ a combination of measures, including the strategies relevant for the period of transition.

## Background

European transformation has catalyzed political, economical, social, and cultural changes in the Western and Eastern Europe during the last decades. A lot of effects on economical and behavioral alcohol related issues were observed due to economical changes of the former Soviet block and due to the European Union enlargement, which has also involved the Baltics and other neighboring countries in transition. Therefore the investigation of alcohol consumption behaviors in the Baltic countries is significant in the European context as well. The Baltic countries suffered from high level of harmful consequences related to extensive use of alcoholic beverages [1-3]. In Estonia, Latvia, and Lithuania alcohol abuse is recognized as a social problem and there is aggregate level evidence on the negative consequences of drinking and alcoholism [4,5].

Epidemiological data describing the trends for alcohol consumption among young people show a continuing rise in the prevalence of drunkenness in some countries of the European region [6,7]. In the recent years the most evident increase was observed in the countries of Eastern and Central Europe [8]. The study on Health Behavior in School-aged Children (HBSC) allows making an international comparison and trend analysis over time [9-11]. The comparison of 1993/94 and 1997/98 survey data showed that 9 out of 23 participating countries had a significant increase in the proportion of 15-year-old students having been drunk two or more times in life, while no decrease was observed [6,12]. Other authors also acknowledge that the rising prevalence of drunkenness could possibly indicate the rise of alcohol consumption among young people on the global scale [8,13].

Estonia, Latvia, and Lithuania are neighboring countries situated on the Eastern shore of the Baltic Sea. For fifty years being part of the former Soviet Union they followed the same model of economy, education, welfare, and health care. They share many typical Eastern European characteristics [2,14,15]. A decade of transition has already eroded many soviet doctrines once closely held in the region, and this dissolution was liberating for the countries. However, the period of transition made young people susceptible to behavioral risks and other potential dangers [16,17].

Although there is an increasing need for information on risk factor levels and health behavior patterns in the former Eastern Europe, very few studies have thoroughly collected and standardized comparative data. In addition, the trends have been analyzed far less than the cross-sectional differences. Such analyses have been already performed among adult population of the Baltic countries, based on FinBalt Health Monitor study [3,18-20] and other national reports [21-23]. Moreover, there has been very little research and detailed discussion on the role of the changing socio-economic environment for health behavior of young people in societies going through a period of transition.

This article aims to estimate the prevalence and trends of alcohol consumption and drunkenness among adolescents of Estonia, Latvia, and Lithuania in 1993/94, 1997/98, and 2001/02. The research analysis is based on the data of three HBSC surveys. The study has focused on the following objectives:

▪ To compare the prevalence and trends of alcohol consumption and drunkenness among students in Estonia, Latvia, and Lithuania over the period of transition.

▪ To assess the relationships between the perceived family economic status and the consumption of alcohol among the students of Estonia, Latvia, and Lithuania in 1993/94, 1997/98, and 2001/02.

## Methods

### Samples and survey procedures

Our research analysis represents the part of HBSC study, which was initiated by five European countries in 1982 and recently involves the target population from 35 countries or regions, under the auspices of the WHO Regional Office for Europe. The comprehensive surveys of national representative samples of 11, 13, and 15-year-old adolescents are conducted every four years. The last three surveys of 1993/94, 1997/98, and 2001/02 school year were carried out in all three Baltic countries. The standardized cross-national research methodology, which was developed by the team of experts, was used [24-26].

In each country, a cluster sampling design was applied. School classes were used as sampling units. Samples of students were drawn to be representative by age and gender. Recommended sample sizes for each country were about 1500 students per age group [25].

Every effort was made to ensure that the HBSC protocol was followed and that the survey instruments, data collection, and processing procedures were consistent. Each participating country obtained approval to conduct the survey from the ethics review board or equivalent regulatory body associate with the institution conducting each national survey. Specially trained personnel, teachers, and school nurses administered the completion of questionnaires in school classrooms. The questioning was anonymous. Response rates were over 90%. Upon the completion of the fieldwork, the data were prepared using standard documentation and submitted to the HBSC International Data Bank at the University of Bergen, Norway. The data were checked, cleaned, and returned to the countries for further statistical processing. The investigation was conformed to the principles outlined in the Declaration of Helsinki and approved by ethics committees in three countries.

The present analysis is based on 5287 boys and 6485 girls (total 11772) aged 15 from Estonia, Latvia, and Lithuania surveyed in 1993/94, 1997/98, and 2001/02 school year (Table 1). We have restricted our analyses to 15-year-olds because this age group represents the diversity of behavioral problems in adolescents better than younger school-aged children [12,27].

### Questionnaire and variables

Questionnaire topics and items for HBSC surveys were discussed among the national research teams and finally were selected at the international project meetings [9-11,24-26]. The national questionnaires were approved after their translation from the standard English version into the national language and later followed by an independent retranslation into English.

The questionnaires have included a few questions regarding alcohol consumption. The two questions on alcohol consumption, which wording was retained in each survey, are described and discussed in this research article.

The frequency of alcohol consumption was investigated by asking Question 1: *At present, how often do you drink alcoholic beverage: a) beer; b) wine/sparkling wine; c) spirits/liquor? *The following multiple-choice answers were included: a) *every day*; b) *every week*; c) *every month*; d) *rarely*; e) *never*.

Drunkenness has been assessed with Question 2: *Have you ever had so much alcohol that you were really drunk? *The following multiple-choice answers were offered: a) *no, never*; b) *yes, once*; c) *yes*, *2–3 times*; d) *yes*, *4–10 times*; e) *yes*, *more than 10 times*.

The respondents were assigned to the following groups according to the frequency of alcohol consumption: a) not users of alcohol (answered "never used" for all three categories of alcoholic beverages); b) regular users (used beer, wine/sparkling wine or spirits/liquor – "every week" or more often); and c) irregular users (drank anything alcoholic "every month" or less frequently).

Self-reports on drunkenness provide a measure of excessive alcohol use. The proportions of those who claimed having been drunk once and who reported having been drunk twice or more are presented in this paper as cut-off points [6].

Over the years, the HBSC study group has developed several Family Affluence Scales for measuring socio-economical status of the family [11]. Therefore the scales and questions varied from survey to survey. Due to this fact it was impossible to use the findings for our international comparison. Instead of these scales we applied more universal question that was designed to measure young people's perception of their own family's socio-economic circumstances for all three surveys:*How well off do you think your family is? *Response options were: a) *very well off*; b)*quite well off*; c)*average*; d)*not so well off*; e)*not at all well off*. In analyses, the first two categories were recoded into *high wealth*, and the last two categories were recoded into *low wealth*.

### Statistical analysis

The data were analyzed using the statistical package SPSS (version 11.5). Statistical hypotheses were evaluated by Z and χ^2 ^tests. The level of statistical significance was established as p < 0.05. In order to obtain an appropriate statistical significance the data from three surveys were combined. For this reason, the data in groups of respondents selected by country, gender and family's wealth were weighted seeking to obtain equal sample size for each year of the survey. The odds of consuming alcoholic beverages weekly according to the family wealth were estimated using multiple logistic regression analyses with adjustment for the year of the survey as a categorical covariate. Due to the fact that gender is a strong determinant of drinking rates, the results for boys and girls were analyzed separately.

## Results

### Prevalence of drinking beer, wine, and spirits

Table 2 presents the distribution of students' answers about regular (at least weekly) use of different alcoholic drinks (beer, wine, spirits/liquor) in 1993/94, 1997/98, and 2001/02 by gender and by country. It was established that beer was the most popular alcoholic beverage used in all three Baltic countries. The data also showed a statistically significant increase of beer consumption during eight years of observation both among boys and girls. However the trend curve was different for Latvian boys: the rate of regular consumption of beer was increasing sharply during the first sub-period of observation (from 16.1% to 25.5%) while in the second sub-period this rate went down (to 17.1%) and almost reached the previous level.

The proportion of students who have reported regular use of wine and spirit/liquor was low in 1993/94. However a significant increase in the number of wine among boys and spirits among boys and girls was recorded during eight years of observation in Estonia. Similar but not statistically significant rising trend was also noticed among adolescents in Latvia and Lithuania (with an exception of wine consumption among Latvian boys).

### Prevalence of regular and irregular drinking

Table 3 describes the prevalence of regular (weekly or more frequently) and irregular (less frequently than weekly) use of anything alcoholic across the Baltic countries among 15-year-olds in the surveys of 1993/94, 1997/98, and 2001/02.

During the period of the study the total percentage of students, who reported regular and irregular consumption of alcohol, has increased statistically significantly in Estonia (p < 0.05), but remained steady in Latvia and Lithuania. However, the regular use of alcohol has almost doubled in boys and in girls alike. The data analysis of the last survey showed that about 25% of boys and 12.5% of girls were using alcohol at least weekly. However, Latvian boys were an exception due to the lower regular consumption of alcohol in 2001/02.

The use of alcohol was also compared among boys and girls. The overall percentage in boys and girls who reported currently consuming alcohol (regularly and irregularly) did not differ significantly across all the Baltic countries in different surveys. The percentage of regular alcohol consumption in boys was about twice higher. However, irregular drinking of alcoholic beverages was more prevalent among girls than among boys in the relevant countries and surveys.

### Prevalence of drunkenness

Table 4 presents the distribution of students' answers to the question "Have you ever had so much alcohol that you were really drunk?" Across the three countries, boys gave a positive answer to this question more frequently than girls. However, the girls have reported similar frequency of single drunkenness episode as boys.

The data presented provide the evidence that significant increase in the prevalence of drunkenness was observed during the study period. On average, 30% of boys and 15% of girls in 1993/94 and 52% of boys and 36% of girls in 2001/02 reported two or more episodes of drunkenness. Over the eight year study period the rates of multiple episodes of drunkenness have more than quadrupled in Estonian girls, and more than doubled in Estonian boys and in Lithuanian boys and girls.

### Consumption of alcohol according to the perceived wealth of the family

The complete data on socioeconomic status of the family was absent due to non-reporting for 2% of respondents from three countries. The rating of perceived family wealth among 15-year-old students from the Baltic countries differed significantly. In 1993/94 the percentages of low family wealth were 38.2%, 11.6%, and 12.7% in Lithuania, Latvia, and Estonia respectively. Therefore, the percentage of such families has decreased and the differences between the countries have diminished over the studied period.

Table 5 demonstrates how the frequency rate of regular alcohol consumption among students depends on the perceived wealth of their families. There is evidence among boys and girls that students from the families perceived by them as wealthy are more likely to drink weekly than students from the families perceived by them as not wealthy. Significant trends in shapes of the regular alcohol consumption prevalence by categories of family wealth were marked in data from distinct surveys in all the Baltic countries with an exception of the girls from Lithuania. As the numbers of respondents who used alcohol regularly within the categories of family wealth were small, the findings of the three surveys were combined. The gradient was statistically significant among the boys from Latvia and Lithuania, and among the girls from Estonia.

The associations between regular drinking and family wealth were explored in a model of logistic regression. In this model, odds ratios within the categories of family wealth were calculated with and without adjustment for years of the survey (Table 6). Considering both methods, the odds of consuming alcohol at least once a week tended to be higher among students in higher family wealth categories. Statistically significant values of odds ratio adjusted for the years of the survey were marked among the boys from all the Baltic countries and among the girls from Estonia.

## Discussion

This research was part of the international HBSC study [9-11]. Therefore, the presented analysis is the first attempt to compare data on the consumption of alcohol between adolescents from Estonia, Latvia, and Lithuania with respect to other European countries and in historical perspective of eight years of the study. This was partially achieved due to significant attention paid to the issues of validity in this survey. The response rates were rather high in the three Baltic countries, therefore there is less probability of the prevalence rates underestimation.

The data on trends of alcohol use among adolescents in the Baltic countries have not been presented extensively before although some smaller scale publications were available in these countries [12,28-31]. This health issue, however, was on the agenda of the developed industrial countries for a few decades. Researchers from the United States and European countries have found high prevalence in the consumption of alcohol without significant increase during the last few decades in different groups of young people [7,32]. However, the rise in alcohol consumption during the last decades was reported in Finland [33] and some other European countries [8].

The results of our study indicated the increase of alcohol consumption among young people in the Baltic countries over the period between 1993 and 2002. We compared how the prevalence of drunkenness was changing in 15-year-old students of the Baltic countries during the study period in European context. In 1993/94 Denmark, Wales, Scotland, and Finland were among the countries, which exceeded the limit of 50 percent according to our criteria of drunkenness ("two and more times") for boys and girls. Out of 25 countries or regions participating in the survey Latvia took the 12^th ^place, Lithuania took the 18^th ^place, and Estonia took the 20^th ^place in ranking order by descending frequency rate of drunkenness estimated as a mean for boys and girls [9]. The HBSC survey conducted in 2001/02 reaffirmed that Denmark, Wales, Scotland, and Finland exceeded the drunkenness frequency limit of 50%. Greenland and England were included into this group of countries too. Lithuania and Estonia went down to number 8 and 9, respectively out of 35 countries that had conducted the HBSC survey. Latvia retained the middle position and was rated as number 20 [11]. The corresponding figures for the 15-year-olds were also obtained for other indicators of alcohol consumption. These findings suggested that young people from the Baltic countries had a tendency to integrate among their peers from those West European and Nordic countries (Denmark, Wales, Scotland, Finland, Greenland, England), where alcohol consumption among the students is high.

We have compared the methods used and our results with other cross-national health behavior surveys. ESPAD (European School Survey on Alcohol and other Drugs) conducted in 1995, 1999, 2003 with the aim to investigate the consumption of alcohol, drugs, and tobacco among 15–16-year-old students is similar but at the same time much more specialized [8]. The Baltic countries were also involved in this survey among 35 other European countries. The set of ESPAD questions is rather different from HBSC questionnaire: this research instrument included more items on each separate behavioral problem (alcohol, drugs, and smoking). Despite some methodological differences, the comparison of results from both studies allowed having a deeper scientific insight into the problem when comparing the trends.

Alcohol use 20 times or more during the last 12 months is a measure from the ESPAD study that could be considered as the most comparable to regular alcohol drinking measure in our study. According to the ESPAD report [8], an increase of the proportion of students who drank alcohol in this regularity was observed in a large number of countries, mainly in the Eastern Europe. Over the years from 1995 to 2003, Estonia and Lithuania (data for Latvia in 1995 are missing) were among those countries where a continuously increasing proportion of students reported drinking 20 times or more within the last 12 months.

The ESPAD survey of 2003 reports that the majority of the 15–16-year-olds have been drunk at least once in their lifetime in 30 countries out of 35 studied [8]. Moreover considering the changes between the surveys of 1995 and 2003, the proportion of students who have been drunk 20 times or more in a lifetime in 12 out of 28 countries increased considerably, while the decrease was observed only in one of the participating countries [8]. Two of the three Baltic countries, Estonia and Lithuania, were included into the group of countries, where unidirectional increase in the proportion of students, who reported this behavior, was observed over the years. This finding of the ESPAD study significantly correlates with the results of our study showing a particular increase in the prevalence of drunkenness among Estonian and Lithuanian students. It is also important to note that both the HBSC and the ESPAD studies identify Denmark as the country where the proportion of students who reported having been drunk remained in the highest rank over the studied period.

In general the comparisons between the HBSC and the ESPAD surveys demonstrate very similar results in trends of alcohol consumption among students in the Baltic States during the last decade. Therefore it is more difficult to draw inferences on the other countries of Central and Eastern Europe because of less evident similarities between the HBSC and the ESPAD surveys conducted in these countries.

The Baltic countries are considered an example of regional homogeneity, especially when taking into consideration historical parallels of the last century, economical indicators, and similar growth of the national economies. The Baltic economies grew by almost half from their initial level during 1996–2003: cumulative growth was 51% for Estonia, 59% for Latvia, and 52% for Lithuania [34]. However, the official statistics and research surveys have already proved that there is non-homogeneity in the area of health and behaviors in the Baltic countries [2,18-20]. It allowed developing the hypothesis regarding possible difference of health behavior, which is the outcome of economical, social, and cultural differences. The market also plays an important role in establishing health behaviors, such as the use of alcohol. Although on the aggregate level of the country it is impossible to assess the impact of particular factors on the patterns recorded, taking into account the changes of lifestyle and the policies related to alcohol might facilitate the interpretation of these data.

Obviously, eight years is a relatively short period for the assessment of the changes in national lifestyles. However, these years in the Baltic countries were of particular interest due to political and economic situation. The period of transition has opened up borders, has changed the values and opportunities and has begun the process accompanied by stress and turmoil. Following the privatization and economic liberalization alcohol and tobacco industry in the region started marketing campaigns aiming to increase the production and sales. The recorded annual consumption of pure alcohol according to the official statistics varied from 8 to 12 liters per capita over 1994–2000 [35,36]. The extent of unrecorded consumption of alcohol was also significant [37].

One of the unfortunate consequences of transition period is the increased willingness of many young people to experiment with legal and illegal drugs, while these drugs become more easily available. Branding and advertising associate alcohol consumption and smoking with an affluent and advantaged western lifestyle or with other images, which are directly appealing to young people. Over the last decade the advertising of alcohol (except beer in Latvia) was restricted by law across all the Baltic countries, particularly for broadcast media. Age limit for purchasing alcoholic beverages in a bar or in a shop is 18 years. Although all the Baltic countries have reported partial restriction on alcohol (mostly spirits) consumption in public places, beer marketing is on the increase in sport events and pop music concerts, targeting young people [38].

It is evident that globalization of culture sometimes has different behavioral effects in young people. Also more liberal and open marketing of alcohol, tobacco products could have the negative consequences [7,39]. In our research alcohol consumption behavior was assessed in the broader international context in order to evaluate the process of integration of the Baltic countries to the European economical and cultural space. The data of three HBSC surveys in sequence demonstrated that young people from the Baltic countries were consuming the quantities of alcohol which were drawing them closer to some of the western countries (Denmark, UK, Finland, etc.), characterized by relatively high consumption of alcohol in young generation. Assimilation of lifestyles between the adolescents from Eastern, Central and Western Europe has been detected regarding smoking, drug use, and other habits as well [12].

The evidence is growing that the lifestyle is also influenced by behavioral patterns common to a person's social group and by more general socio-economic conditions [39]. In the majority of the developed European countries material welfare has increased in a rather limited, socially and economically privileged, population group, which was better placed to adopt health promoting changes in behavior [40]. Such assumption raised an important objective for our research – to test the relationship between alcohol consumption and family economic status in young people from the Baltic countries.

Evidence about the relationship between socio-economic status and health risk behaviors in adolescence, however, is often inconsistent or even contradictory. The picture gets even more complex with the introduction of differences between male and female adolescents [41]. Our study has demonstrated that students from the wealthy families were more likely to drink alcoholic beverages weekly than students from the poorer families (significant associations were found among the boys from all the Baltic countries and among the girls from Estonia). Similar results for Lithuania and Estonia (data for Latvia were missing) as well as for some other countries were presented in the ESPAD report 2003 [8].

These findings could be explained by the recently popular term – "the diseases of affluence" – which relates to higher levels of noncommunicable disease risks due to improved access of population for consumption of unhealthy food (alcohol, diet rich in fat and carbohydrates) and use of industrialized services [42]. This phenomenon is apparently common in the developed societies, but could be also related to some specific socio-economic periods of transition such as in the Baltic countries influencing health behavior of adolescents.

Given the increasing prevalence of alcohol use among youth in the Baltic countries considerable efforts should be directed towards identifying effective preventive measures. The main implication of our findings is that the rise of economy in the region does not mean the improvement in risky health behavior of adolescents. The most recent and promising prevention approaches are based on the psychosocial influences that promote alcohol use initiation by increasing an awareness of the social influences, restriction in laws and norms unfavorable towards alcohol use, and building drug resistance skills [43,44]. These approaches are on the agenda of the national health policy but have not been put into practice yet in any of the Baltic countries [1,14].

Data from many recent studies imply that social determinants should be taken into account when conducting intervention programmes, which are aiming to prevent alcohol consumption in youth. The paradigm on "diseases of affluence" also implies that risk factors of non-communicable disease could also have an impact on middle and higher income population groups [42]. Due to this reason such interventions should be appropriately designed for adolescents from higher socio-economic backgrounds and attempt to diminish their positive attitudes towards excessive alcohol consumption. Literature review shows that the most promising preventive strategies to achieve this objective might include the development of parental skills and functional family therapy, social competence skills, use of young leaders as models, youth involvement in alternative leisure activities, etc. [44].

In conclusion, the results of this study indicate that there was an increase of alcohol consumption among young people in the Baltic countries over the period between 1993 and 2002. This has caused young people from the Baltic countries to integrate among their peers from those West European and Nordic countries, where alcohol consumption among students is relatively high. It is also alarming that the risk of alcohol consumption among young people in the Baltic region was linked to the higher wealth of the family. These results of the study raise important policy implications for the development of alcohol abuse prevention programs for youth. A combination of measures should be employed in efforts to deal with this problem, including strategies relevant for the period of transition. National health programs for the Baltic countries aim to reduce alcohol consumption by 25% by the year 2010 [37,45].

## Conclusion

The most popular alcoholic beverage among the students from the Baltic countries was beer. The consumption of this beverage increased more than twice among boys and girls during eight years of observation (with an exception of Latvian boys). In 1993 – 2002, regular use of alcohol and drunkenness increased considerably among boys from Estonia and Lithuania and among girls in all the three Baltic countries. On average, 25% of boys and 12.5% of girls aged 15 in the Baltic countries reported consuming alcoholic beverages at least once a week in 2001/02 school year. The use of alcoholic beverages among students was linked to their family wealth: students from the families perceived by them as wealthy were more likely to drink regularly than the students from the families of lower perceived wealth. Over the period of 1993 – 2002 the increased prevalence of alcohol consumption has caused the fact that young people from the Baltic countries have reached the same level of consumption as in some western European and Nordic countries, where alcohol consumption among students is relatively high. The efforts of dealing with the growing alcohol consumption among young people in the Baltic countries should employ a combination of measures, including the strategies relevant for the period of transition.

## Competing interests

The author(s) declare that they have no competing interests.

## Authors' contributions

AZ is the principal investigator of the HBSC study in Lithuania. He has contributed to the design of the study, statistical analysis, and interpretation of the data and has drafted the paper. LS has contributed in acquisition, interpretation of the data and has aided in preparation, revision of the paper. MM is the principal investigator of the HBSC study in Estonia. She has contributed to the design of the study and the interpretation of the data, revision of the paper. IP is the principal investigator of the HBSC study in Latvia. She has contributed to the design of the study, participated in interpretation of the data and the critical revision of the paper for the importance of the intellectual content. All the authors have read and approved the final draft of the paper.

## Pre-publication history

The pre-publication history for this paper can be accessed here:



## Figures and Tables

**Table 1:** Number of respondents, response rate, and date of fieldwork by gender and country in 1993/04, 1997/98, and 2001/02

Year of the survey, country	Number of respondents			Response rate	Date of fieldwork
			
	Boys	Girls	Total		
***1993/94***					
Estonia	549	630	1179	92%	February, 1994
Latvia	472	791	1263	92%	November-December, 1993
Lithuania	788	971	1759	94%	March-April, 1994

***1997/98***					
Estonia	252	335	587	90%	February-March, 1998
Latvia	508	757	1265	94%	November-December, 1997
Lithuania	636	799	1435	95%	March-April, 1994

***2001/02***					
Estonia	619	648	1267	94%	October-November, 2001
Latvia	481	631	1112	94%	November-December, 2001
Lithuania	982	923	1905	96%	March-April, 1994

***Total***	**5287**	**6485**	**11772**		

**Table 2:** Percentage of students who regularly consumed beer, wine, and spirits by gender and country in 1993/94, 1997/98, and 2001/02

Type of alcoholic drinks, country	Boys			Girls		
	
	1993/94	1997/98	2001/02	1993/94	1997/98	2001/02
***Beer***						
Estonia	11.6	19.3	26.8*	2.4	8.1	6.5*
Latvia	16.1	25.5	17.1	3.2	5.6	7.4*
Lithuania	12.1	15.9	26.7*	4.7	8.1	10.2*

***Wine/sparkling wine***						
Estonia	3.4	3.8	6.5*	2.7	3.8	4.9
Latvia	6.4	5.9	4.8	2.8	5.5	4.3
Lithuania	2.0	2.9	3.1	2.1	2.3	3.3

***Spirits/liquor***						
Estonia	3.1	3.8	8.9*	1.0	1.0	5.1*
Latvia	4.0	6.4	4.3	3.3	2.9	4.2
Lithuania	3.3	1.3	5.1	1.2	0.4	1.6

* statistically significant difference between the rate of the answer in 1993/94 and 2001/02 (Z test, p < 0.05).

**Table 3:** Percentage of regular and irregular drinkers of alcohol by gender and country in 1993/94, 1997/98, and 2001/02

Gender, country	Regularity of alcohol use								
	
	Regular			Irregular			Total		
	
	1993/94	1997/98	2001/02	1993/94	1997/98	2001/02	1993/94	1997/98	2001/02
***Boys***									
Estonia	14.7	23.5	28.1*	64.1	57.1	56.4*	78.8	85.2	84.5*
Latvia	19.9	29.4	19.8	63.6	62.8	65.7	83.5	92.2	85.5
Lithuania	15.7	18.1	27.6*	72.2	69.3	60.1*	87.9	87.4	87.7

***Girls***									
Estonia	5.2	11.5	12.0*	69.8	73.7	70.9	75.0	85.2	82.9*
Latvia	7.7	12.6	12.6*	79.0	75.3	70.1*	86.7	87.9	82.7
Lithuania	6.8	10.5	13.0*	82.1	77.6	75.5*	88.9	88.1	88.5

* statistically significant difference between the rate of the answer in 1993/94 and 2001/02 (Z test, p < 0.05).

**Table 4:** Percentage of students who have been really drunk by gender and country in 1993/94, 1997/98, and 2001/02

Gender, country	Have been really drunk								
	
	Once			Twice or more			Total		
	
	1993/94	1997/98	2001/02	1993/94	1997/98	2001/02	1993/94	1997/98	2001/02
***Boys***									
Estonia	21.1	16.0	17.6	25.9	44.0	56.7*	47.0	60.0	74.3*
Latvia	23.4	23.5	22.8	35.2	47.0	41.4*	58.6	70.5	64.2*
Lithuania	22.9	26.6	20.5	27.3	32.3	56.7*	50.2	58.9	77.2*

***Girls***									
Estonia	15.7	16.7	17.8	9.7	23.0	42.0*	25.4	39.7	59.8*
Latvia	24.6	22.5	27.3	20.5	23.2	25.4*	45.1	45.7	52.7*
Lithuania	21.6	25.2	26.7*	16.5	20.4	42.2*	38.1	45.6	68.9*

* statistically significant difference between the rate of the answer in 1993/94 and 2001/02 (Z test, p < 0.05).

**Table 5:** Students consuming alcohol at least weekly by gender, country, year of the survey, and level of family wealth

Gender, country	Level of family wealth	Drank alcoholic beverages at least weekly, n (%)			
		
		1993/94	1997/98	2001/02	Total with weighted frequency
***Boys:***					
Estonia	Low	5 (8.1%)	4 (12.1%)	13 (27.7%)	22 (16.0%)
	Average	58 (17.2%)	45 (26.5%)	122 (25.3%)	225 (23.0%)
	High	10 (11.2%)	4 (15.4%)	39 (44.8%)*	53 (23.8%)

Latvia	Low	2 (3.9%)	12 (18.8%)	7 (18.4%)	21 (13.7%)
	Average	42 (17.6%)	69 (32.1%)	24 (15.9%)	135 (21.9%)
	High	47 (27.6%)*	59 (30.6%)*	61 (22.2%)	167 (26.8%)*

Lithuania	Low	24 (9.7%)	18 (14.8%)	69 (24.3%)	111 (16.3%)
	Average	66 (19.2%)	64 (19.9%)	138 (26.3%)	268 (22.5%)
	High	12 (20.0%)	18 (16.5%)	61 (38.1%)*	91 (24.9%)*

***Girls:***					
Estonia	Low	4 (4.1%)	6 (9.1%)	5 (9.6%)	15 (7.6%)
	Average	14 (3.7%)	20 (10.1%)	62 (12.0%)	96 (8.6%)
	High	12 (13.2%)*	8 (21.6%)	11 (14.3%)	31 (16.4%)*

Latvia	Low	14 (9.0%)	7 (6.4%)	12 (13.0%)	33 (9.5%)
	Average	27 (6.9%)	47 (13.1%)	29 (12.6%)	103 (10.9%)
	High	20 (9.3%)	32 (14.2%)*	37 (12.5%)	89 (12.0%)

Lithuania	Low	20 (5.6%)	22 (10.0%)	47 (12.6%)	89 (9.4%)
	Average	30 (8.0%)	40 (11.1%)	56 (13.5%)	126 (10.9%)
	High	9 (12.3%)	12 (9.5%)	15 (12.2%)	36 (11.3%)

* statistically significant difference between the rate of answers in groups of family wealth (χ^2 ^test, p < 0.05).

**Table 6:** Odds ratios (OR) for likelihood of consuming alcohol at least weekly according to the level of family wealth by country and gender

Gender, country	Level of family wealth	Odds ratio, crude		Odds ratio adjusted for year of the survey	
		
		OR	95% CI	OR	95% CI
***Boys:***					
Estonia	Low	1	-	1	-
	Average	1.53	0.96 – 2.45	1.76*	1.04 – 2.99
	High	1.67	0.97 – 2.89	1.59	0.85 – 2.99

Latvia	Low	1	-	1	-
	Average	1.76*	1.07 – 2.90	2.07*	1.33 – 3.79
	High	2.33*	1.42 – 3.81	2.82*	1.55 – 5.12

Lithuania	Low	1	-	1	-
	Average	1.42*	1.11 – 1.82	1.52*	1.17 – 1.99
	High	1.72*	1.24 – 2.37	1.72*	1.22 – 2.44

***Girls:***					
Estonia	Low	1	-	1	-
	Average	1.14	0.66 – 1.96	1.09	0.60 – 1.97
	High	2.37*	1.27 – 4.40	2.49*	1.29 – 4.81

Latvia	Low	1	-	1	-
	Average	1.15	0.76 – 1.72	1.17	0.76 – 1.79
	High	1.29	0.85 – 1.96	1.34	0.87 – 2.07

Lithuania	Low	1	-	1	-
	Average	1.17	0.88 – 1.56	1.21	0.90 – 1.64
	High	1.23	0.82 – 1.84	1.29	0.85 – 1.96

* statistically significant relation (p < 0.05). CI – confidence interval.
